# Retraction Note: Downregulation of miR-196-5p induced by hypoxia drives tumorigenesis and metastasis in hepatocellular carcinoma

**DOI:** 10.1007/s12672-023-00659-6

**Published:** 2023-04-24

**Authors:** Hao Zheng, Feng-rui Bi, Yuan Yang, Yong-gang Hong, Jun-sheng Ni, Long Ma, Ming-hua Liu, Li-qiang Hao, Wei-ping Zhou, Li-hua Song, Hong-Li Yan

**Affiliations:** 1 Department of Reproductive Heredity Center, Changhai Hospital, Second Military Medical University, Shanghai, 200433 People’s Republic of China; 2 Third Department of Hepatic Surgery, Eastern Hepatobiliary Surgery Hospital, Second Military Medical University, Shanghai, 200438 People’s Republic of China; 3 Key Laboratory of Signalling Regulation and Targeting Therapy of Liver Cancer (SMMU), Ministry of Education, Shanghai, 200438 People’s Republic of China; 4 Shanghai Key Laboratory of Hepatobiliary Tumor Biology (EHBH), Shanghai, 200438 People’s Republic of China; 5 Department of Colorectal Surgery, Changhai Hospital, Second Military Medical University, Shanghai, 200433 People’s Republic of China; 6 School of Agriculture and Biology, Shanghai Jiao Tong University, Shanghai, 200240 People’s Republic of China

**Retraction: Discover Oncology (2019) 10:177–189** **https://doi.org/10.1007/s12672-019-00370-5**

The Editor-in-Chief has retracted this article. The authors contacted the journal after discovering they had used the incorrect data for Figure 3A, 3B, Figure 4C, 4I, Figure 6A, 6G, 6H and Figure S2. The Editor-in-Chief has therefore lost confidence in the reliability of the results presented in this article. The authors have been offered to submit a revised manuscript for further peer review. None of the authors agree to this retraction.

